# Supplementing yeast culture to beef heifers consuming a forage-based diet

**DOI:** 10.1093/tas/txaf103

**Published:** 2025-08-19

**Authors:** Autumn T Pickett, Reinaldo F Cooke, Izadora S de Souza, Willian A de Souza, Guilherme A Monteiro, Mykael B do Prado, Vinícius N Gouvêa, Rafael C Araujo, Shea J Mackey

**Affiliations:** Department of Animal Science - Texas A&M University, College Station, TX 77843, USA; Department of Animal Science - Texas A&M University, College Station, TX 77843, USA; Department of Animal Science - Texas A&M University, College Station, TX 77843, USA; Department of Animal Science - Texas A&M University, College Station, TX 77843, USA; Department of Animal Science - Texas A&M University, College Station, TX 77843, USA; Texas A&M AgriLife Research and Extension Center, Amarillo, TX 79106, USA; Texas A&M AgriLife Research and Extension Center, Amarillo, TX 79106, USA; GRASP Indústria e Comércio Ltda., Curitiba, PR 81260-000, Brazil; Department of Animal Science - Texas A&M University, College Station, TX 77843, USA

**Keywords:** beef heifers, forage, rumen, yeast culture

## Abstract

This experiment evaluated the effects of supplementing yeast culture (**YC;***Saccharomyces cerevisiae*) on in situ ruminal degradability, rumen fermentation and microbiota responses of heifers consuming a forage-based diet. Twelve ruminally-cannulated Angus-influenced heifers were ranked by body weight (**BW;** 180 ± 4 kg) and assigned to 4 groups of 3 heifers each. Groups were enrolled in a replicated 3 × 3 Latin square design containing 3 periods of 21 d and 14-d washout intervals. Groups were assigned to receive no YC (**CON**), or 1.5 g of YC/100 kg of heifer BW (**YC1.5**) or 3.0 g of YC/100 kg of heifer BW (**YC3.0**). During each period, heifers (n = 12/treatment) were housed in individual pens and offered water and bermudagrass hay (*Cynodon dactylon*) for ad libitum consumption, in addition to 650 g/d (as-fed basis) of a corn-based concentrate. The YC was mixed daily with the concentrate during each period (d 0 to 21). Ruminal in situ disappearance of forage dry matter (**DM**) and neutral detergent fiber (**NDF**) were evaluated by incubating polyester bags with 4 g of dietary hay on d 17 for 96 h. Rumen fluid samples were collected on d 0, 8, and 16, in a manner that the final collection (d 16) did not interfere with in situ procedures on d 17. Apparent total-tract digestibility (**aTTD**) of nutrients was calculated using fecal samples collected every 12-h from d 17 to 21. Data were analyzed using orthogonal contrasts (linear and quadratic) with heifer as the experimental unit. Feed intake was not affected (*P *≥ 0.14) by treatments. Inclusion of YC linearly increased (*P *≤ 0.04) propionate and iso-butyrate concentrations in the rumen fluid, and tended (*P *≤ 0.09) to increase acetate and total volatile fatty acid (**VFA**) concentrations. Inclusion of YC linearly decreased (*P *= 0.03) the relative abundance of the bacterial genus *Succiniclasticum*, and linearly increased (*P *= 0.04) genera Shannon diversity index in the rumen fluid. Inclusion of YC linearly increased (*P *≤ 0.05) ruminal disappearance rate of hay DM and NDF, but did not affect (*P *≥ 0.40) degradability of DM and NDF. Inclusion of YC linearly increased (*P *≤ 0.05) aTTD of starch and NDF. Inclusion of YC linearly increased (*P *= 0.04) heifer average daily gain and gain:feed (**G:F**). Collectively, supplementing 1.5 or 3.0 g of YC/100 kg of BW linearly enhanced utilization of dietary nutrients and production of ruminal VFA in beef heifers consuming a forage-based diet.

## Teaser

Yeast products are often used as feed additive for beef cattle, including as dead cells of *Saccharomyces cerevisiae* . Most research conducted to date evaluated this feed additive for feedlot cattle; hence, this study is novel by supplementing yeast products to beef cattle consuming forage-based diets.

## INTRODUCTION

Yeast products are often used as feed additive for cattle (Poppy et al., 2012), particularly as live or dead cells of *Saccharomyces cerevisiae* (AAFCO, 2011). Live yeast products are dried and must contain > 1.5 × 10^10^ CFU/g of live cells (dry matter; **DM**) to provide a probiotic effect to the rumen microbiota (Baker et al., 2022). Yeast cultures (**YC**) are products based on dead cells and their fermentation growth medium, which provides growth-promoting substrates with prebiotic effects to the ruminal flora (Callaway and Martin, 1997; Newbold and Rode, 2006). Nonetheless, Oeztuerk et al. (2005) compared live and autoclaved *Saccharomyces boulardii* cells using an in vitro ruminal microbial metabolism model, and reported no clear advantages when living yeast cells were used. These authors suggested that supplementing yeast products improve rumen microbial metabolism mostly due to the prebiotic effects associated with YC.

Supplemental YC improved performance of feedlot cattle (Baker et al., 2022; Batista et al., 2022), mainly by improving rumen digestibility of DM, crude protein (**CP**), and neutral detergent fiber (**NDF**) but without improving rumen volatile fatty acid (**VFA**) production (Batista et al., 2022). Limited research, however, has evaluated feeding YC to beef cattle consuming forage-based diets. A meta-analysis by Desnoyers et al. (2009) suggested that supplementing yeast products to dairy ruminants altered ruminal fermentation according to dietary forage content. The benefits of yeast supplementation to some rumen fermentation parameters, particularly ruminal pH, were the greatest in high-concentrate diets (Desnoyers et al., 2009). In turn, positive effects of yeast supplementation on VFA concentrations were observed in diets with elevated NDF content (Desnoyers et al., 2009). The growth-promoting substrates of YC can improve rumen VFA production by enhancing the metabolism of rumen microbes, increasing their cellulolytic ability and VFA synthesis from forage-based diets (Harrison et al., 1988). Therefore, research is warranted to characterize the effects of YC inclusion into forage-fed beef cattle, particularly C4 grasses that predominate in tropical/subtropical regions of the US and the world. These forages have elevated NDF content that impair their ruminal digestibility compared with C3 forages (Cooke et al., 2020), whereas feed additives such as YC are warranted to improve utilization of C4 forages by cattle.

This experiment was designed to address this gap in knowledge by evaluating the effects of YC supplementation on in situ ruminal degradability, rumen fermentation, and ruminal microbiota responses of beef heifers consuming a C4 forage-based diet. Two levels of supplemental YC were evaluated, as we hypothesized that dietary YC inclusion will linearly improve rumen function of forage-fed beef cattle given its prebiotic benefits to the ruminal flora (Callaway and Martin, 1997; Newbold and Rode, 2006). An in situ methodology was adopted as an initial approach to test this hypothesis, and serve as foundation for future studies focusing on growth performance and other production traits in beef cattle (Foster et al., 2023).

## MATERIALS AND METHODS

This experiment was conducted at the Texas A&M—Nutrition & Physiology Center (College Station, TX). All animals were cared for in accordance with acceptable practices and experimental protocols reviewed and approved by the Texas A&M AgriLife Research, Agriculture Animal Care and Use Committee (#2021-0308).

### Animals and Treatments

Twelve ruminally-cannulated yearling, nulliparous, non-pregnant Angus (75%) × Brahman (25%) heifers were used. Heifer unshrunk BW was recorded for three consecutive days prior to the beginning of the experiment, and averaged to represent pretrial BW (180 ± 4 kg). Heifers were ranked by pretrial BW and assigned to 4 groups of 3 heifers each, and groups had equivalent pretrial BW. Groups were enrolled in a replicated 3 × 3 Latin square design containing 3 periods of 21 d, and a 14-d washout interval between periods. Periods of 21 d or less have been used in ruminant nutrition research (Harvatine and Allen, 2006; Mohammed et al., 2014; Batista et al., 2024), whereas rumen fermentation patterns and bacterial community composition days are stabilized 7 d after treatment administration in nutritional experiments with cattle fed tropical forage-based diets (Machado et al., 2016). Heifers were housed in an enclosed barn in individual pens (2 × 4 m) during each period (d 0 to 21). Heifers were maintained as a single group in a drylot pen (20 × 20 m) for the initial 10 d of the washout intervals, and returned to individual pens for the remaining 4 d. Throughout study periods and washout intervals, heifers had ad libitum access to water and bermudagrass hay (*Cynodon dactylon*) and were fed 650 g/d (as-fed basis) of a concentrate (450 g of corn, 180 g of cottonseed meal, and 20 g of vitamin + mineral mix; Table 1) at 0700 h. Hay and concentrate were offered in separate feed bunks. Formulation and feeding rate of the concentrate was selected to yield an average daily gain (**ADG**) of approximately 550 g/d (NASEM, 2016), according to nutritive value of the forage used herein (Table 1).

**Table 1.** Nutrient profile (dry matter basis) of feed ingredients offered to heifers^1^

Item	Hay	Corn	Cottonseed meal
Total digestible nutrients, %	57.0	90.0	70.0
Net energy for maintenance,^2^ Mcal/kg	1.10	2.29	1.63
Net energy for gain,^2^ Mcal/kg	0.53	1.58	1.03
Crude protein, %	9.50	9.20	47.9
Starch, %	4.81	67.3	8.44
Acid detergent fiber, %	34.9	3.30	16.2
Neutral detergent fiber, %	69.5	10.9	26.3
Lignin, %	4.90	1.15	0.600

^1^Analyzed via wet chemistry procedures by a commercial laboratory (Dairy One Forage Laboratory, Ithaca, NY). Heifers had ad libitum access to bermudagrass hay (*Cynodon dactylon*) and were fed 650 g/d (as-fed basis) of a concentrate (450 g of corn, 180 g of cottonseed meal, and 20 g of vitamin + mineral mix). The vitamin + mineral mix contained 21% Ca, 0.01% P, 21% NaCl, 0.20% K, 0.10% Mg, 0.045% Cu, 0.001% Se, 0.280% Zn, 220,000 IU/kg of vitamin A, 19,800 IU/kg of vitamin D3, and 3,500 IU/kg of vitamin E (Anipro Xtraperformance Feeds, College Station, TX).

^2^Calculated with the following equations (NASEM, 2016): Net energy for maintenance = (1.37 × ME)—(0.138 × ME^2^) + (0.0105 × ME^3^) – (1.12); Net energy for gain = (1.42 × ME)—(0.174 × ME^2^) + (0.0122 × ME^3^) – (0.165); ME = (0.82 × DE); 1 kg of TDN = 4.4 Mcal of DE. Given that **ME** = metabolizable energy, **DE** = digestible energy, **TDN** = total digestible nutrients calculated based on Weiss et al. (1992).

At the beginning of each period (d 0), groups were assigned to receive (as-fed basis) 1 of 3 treatments: 1) no YC supplementation (**CON;** n = 12), 2) YC supplementation at 1.5 g/100 kg of heifer BW (**YC1.5;** n = 12) and 3) YC supplementation at 3.0 g/100 kg of heifer BW (**YC3.0;** n = 12). The YC supplement used herein contained dead yeast culture of *S. cerevisiae* with growing medium and metabolites (Factor SC, GRASP Indústria e Comércio Ltda., Curitiba, Brazil). The YC was evaluated via optical microscopy (100x magnification; Nikon Instruments, Melville, NY), and yeast cells stained with methylene blue (7220-79-3; Fisher Chemical; Waltham, MA). No live cells are observed whereas dead yeast cells were counted using a Sedgewick Rafter Counting Chamber (Hausser Scientific; Horsham, PA), resulting in a concentration of 2.05 × 10^9^ dead cells/g of growth medium. The proper dose of YC was mixed daily with the concentrate during each period (from d 0 to 20), which was completely consumed within 30 min after feeding. The doses used in this experiment were chosen according to manufacturer’s recommendation and based on Dias et al. (2018), in which the same YC product was fed at ~2.3 g/100 kg of BW to lactating Holstein cows and improved their feed efficiency. No placebo treatment was offered to CON heifers because YC was provided at less than 6 g/heifer daily (0.12% of expected feed intake).

### Sampling and Laboratorial Analysis

#### Heifer BW and intake.

 Heifer unshrunk BW was recorded on d -2, -1, and 0 relative to the beginning of each period and averaged for initial BW. Final BW was calculated by averaging heifer unshrunk BW on d 19, 20, and 21 of each period. Hay intake was evaluated daily from d 0 to 21 of each period by weighing and collecting samples of the offered and non-consumed hay, and drying samples for 96 h at 50 °C in forced-air ovens for DM calculation. Feed intake was calculated according to daily hay and concentrate intake. Heifer ADG was calculated using initial and final BW of each period. Gain to feed ratio (**G:F**) was calculated according to total feed intake and total BW gain of each heifer within each period. Inclusion of YC into the concentrate was adjusted to heifer initial BW at the beginning of each period.

#### Rumen fermentation and microbiota analysis.

 Ruminal fluid samples (approximately 100 mL) were collected by suction strainer (Raun and Burroughs, 1962) on d 0, 8, and 16 of each period, immediately prior to (0 h) and at 4, 8, and 12 h relative to concentrate feeding of the day. The pH of the rumen samples was immediately measured using portable a meter (Traceable™, Thermo Fisher Scientific; Waltham, MA). Rumen samples were strained through 8 layers of cheesecloth for fluid extraction, stored into individual stainless-steel thermoses to maintain both temperature and an anaerobic environment, and then transported to the laboratory for further processing. A 5-mL subsample of each rumen fluid sample was transferred into individual falcon tubes containing 1 mL of 25% metaphosphoric acid and stored at –20 °C on the same day of collection for ammonia and VFA analysis (Cappellozza et al., 2013; Pickett et al., 2022). Another 5-mL subsample of the rumen fluid collected prior to concentrate feeding (0 h) was immediately snapped-frozen in liquid N for microbiota analysis as described by Pickett et al. (2022). Rumen fluid was chosen for microbiota analyses to complement the VFA and ammonia analyses that were also performed using rumen fluid samples. Moreover, ruminal fluid samples were collected on d 16 for VFA and microbiota analysis before introducing Co-EDTA and Dacron bags (d 17 to 21) into the rumen, and also collected on d 8 as intermediary sampling time within each study period.

#### Ruminal forage disappearance.

Immediately before concentrate feeding on d 17 (h 0), Dacron bags (50 ± 10 µm pore size and 10 × 20 cm bag size; Ankom Technology Corp.; Macedon, NY, US) containing 4 g (DM basis) of ground dietary bermudagrass hay (2-mm screen; Wiley Mill, Model 4; Arthur H. Thomas, Philadelphia, PA) were suspended into the ruminal ventral sac of each heifer, and incubated in triplicates for 0, 2, 4, 6, 8, 12, 24, 36, 48, 72, and 96 h. All bags were soaked in warm water (39 °C) for 15 min before ruminal incubation. After ruminal incubation, bags were washed repeatedly with running water until the rinse water was colorless, and subsequently dried for 96 h at 50 °C in a forced-air oven. The 0-h bags were not incubated in the rumen, but were subjected to the same soaking, washing, and drying procedures applied to the ruminally-incubated bags. Dried samples were weighed for residual DM determination, and triplicates were combined and analyzed for NDF (Robertson and Van Soest, 1981) with sodium sulfite and heat stable amylase (Ankom 200 Fiber Analyzer; Ankom Technology Corp.). Effective degradability of hay DM and NDF were calculated fixing ruminal passage rate at 0.035/h (Scarbrough et al., 2006) and the model from Ørskov and McDonald (1979).

#### Rumen liquid dilution rate and volume.

Immediately before concentrate feeding on d 17 (h 0), each heifer was intra-ruminally pulse-dosed with 5 g of Co-EDTA in a 150-mL aqueous solution (Udén et al., 1980) using a stainless-steel probe with a perforated tip. Ruminal fluid samples were collected as previously described immediately prior to (h 0), and at 0, 2, 4, 6, 8, 12, 24, 36, 48, 72, and 96 h relative to Co-EDTA infusion. A 20-mL subsample of each ruminal fluid was stored (-20 °C) for analysis of Co concentration by atomic absorption using an air/acetylene flame (Model 351 AA/AE Spectrophotometer, Instrumentation Laboratory, Inc., Lexington, MA). Ruminal liquid volume and liquid dilution rate were estimated by regressing the natural logarithm of Co concentration against sampling time (Warner and Stacy, 1968). Lippolis et al. (2017)  infused Co-EDTA and incubated Dacron bags in the rumen of beef heifers at the same time, and simultaneously collected rumen fluid samples and retrieved bags within a 24-h period. No concerns regarding disruption of digesta flow or animal behavior were reported, as authors successfully detected treatment effects on rumen liquid dilution rate and in situ forage disappearance according to their hypothesis.

#### Apparent total-tract nutrient digestibility.

 Fecal grab samples were collected from the rectum of each heifer at 12-h intervals, beginning on d 17 prior to concentrate (h 0) feeding until d 21 (h 96). Fecal samples from each heifer were dried for 168 h at 50 °C in a forced-air oven, composited within each period, and used to calculate apparent total-tract digestibility (**aTTD**) of DM, CP, NDF, and starch. The aTTD for each nutrient was calculated using lignin as an internal marker and nutrient concentrations according to feed intake and feces using the following equation (Ferrareto et al., 2015; DM basis): *aTTD (% of nutrient intake) = 100 – [(dietary marker concentration/fecal marker concentration) × (fecal nutrient concentration/ dietary nutrient concentration)].*

Samples of hay and concentrate ingredients were collected prior to the beginning of each period and pooled across periods. Fecal and feed samples were analyzed by wet chemistry procedures for concentrations of CP (method 984.13; AOAC, 2006), acid detergent fiber (**ADF**; method 973.18 modified for use in an Ankom 200 fiber analyzer, Ankom Technology Corp.; AOAC, 2006), NDF using a-amylase and sodium sulfite (Van Soest et al., 1991; modified for use in an Ankom 200 fiber analyzer, Ankom Technology Corp.), lignin (method 973.18 with sulfuric acid modified for use in an Ankom 200 fiber analyzer, Ankom Technology Corp.; AOAC, 2006), and starch (YSI 2700 SELECT Biochemistry Analyzer; YSI Inc., Yellow Springs, OH). The nutritional profile of dietary ingredients are described in Table 1.

### Statistical Analysis

Heifer was considered the experimental unit for all analyses. All data were analyzed with the MIXED procedure of SAS (SAS Inst. Inc., Cary, NC), using heifer(group) and group as random variables. The model statement used for heifer BW, ADG, and G:F contained the effects of treatment and period as independent variable. The model statement used for feed intake and rumen fluid microbiota contained the effects of treatment, day, the treatment × day interaction, and period as independent variable. The specified term for these repeated statements was day, with heifer(treatment × period) as the subject, and autoregressive as covariance structure based on the Akaike information criterion. The model statement used for all other rumen fluid variables contained the effects of treatment, day, hour, all resultant interactions, and period as an independent variable. The specified term for these repeated statements was hour, with heifer(treatment × period × day) as the subject, and autoregressive as covariance structure based on the Akaike information criterion. Results from ruminal fluid variables collected on d 0, and feed intake from d -3 to 0, were averaged and used as independent covariate in each respectively analysis. All results are reported as least square means, or covariately-adjusted least square means when model contained results prior to treatment administration (d 0). Significance was set at *P* ≤ 0.05 and tendencies were determined if *P* > 0.05 and ≤ 0.10. Orthogonal contrasts were tested to determine if YC inclusion (0, 1.5, or 3.0 g/100 kg of BW) yielded linear or quadratic effects. Contrast coefficients were generated using the IML procedure of SAS (SAS Inst. Inc.). If both contrasts were significant (*P *≤ 0.05), the contrast with the greatest F-value is discussed.

## RESULTS

Heifer initial BW did not differ (*P* ≥ 0.34) among treatments as designed (Table 2). Inclusion of YC linearly increased heifer ADG (*P *= 0.04), resulting in a tendency (*P *= 0.09) for a linear increase in final BW (Table 2). Hay and total feed intake (hay + concentrate) were not affected (*P *≥ 0.14) by treatments (treatment × day, *P *≥ 0.44), whereas G:F was linearly increased (*P *= 0.04) by YC inclusion. Hay and total feed intake were also evaluated from d 8 to 21, considering d 0 to 7 as potential adaptation period (Machado et al., 2016). No treatment effects or treatment × day interaction were detected (*P *≥ 0.18) for hay intake (4.42, 4.54, and 4.42 kg/d for CON, YC1.5, and YC3.0, respectively; SEM = 0.14) or total feed intake (5.02, 5.14, and 5.02 kg/d for CON, YC1.5, and YC3.0, respectively; SEM = 0.14).

**Table 2.** Growth performance of rumen-cannulated forage-fed heifers receiving no yeast culture (**YC**) supplementation (**CON**; n = 12), or YC at (as-fed basis) 1.5 g/100 kg of heifer BW (**YC1.5;** n = 12) or 3.0 g/100 kg of heifer body weight (**YC3.0,** n = 12)^1^^,^^2^

					Contrasts (*P*-value)	
Item	CON	YC1.5	YC3.0	SEM	Linear	Quadratic
Initial BW, kg	184.5	185.5	184.5	4.1	0.98	0.34
Final BW, kg	195.5	197.7	197.9	4.5	0.09	0.41
ADG, kg/d	0.522	0.578	0.635	0.049	0.04	0.99
Feed intake, kg/d						
Hay only	4.33	4.50	4.34	0.14	0.82	0.15
Hay + concentrate	4.93	5.10	4.94	0.15	0.94	0.14
Gain to feed, kg/kg	0.103	0.112	0.125	0.009	0.04	0.85

^1^Heifers were assigned to a 3 × 3 Latin square design, containing 3 periods of 21 d each and a washout interval of 14 d between periods. The YC contained dead yeast culture of *Saccharomyces cerevisiae* with growing medium and metabolites (Factor SC, GRASP Indústria e Comércio Ltda., Curitiba, Brazil), with 1.9 × 10^9^ dead cells/g of growth medium and no presence of live cells (Dias et al., 2018).

^2^Initial body weight (**BW**) was calculated by averaging heifer BW on d -2, -1, and 0 relative to the beginning of each period. Final BW was calculated by averaging heifer BW on d 19, 20, and 21 of each period. Hay intake was evaluated daily, and heifers entirely consumed the concentrate within 30 min after feeding (0700 h). Heifer average daily gain (**ADG**) was calculated using initial and final BW of each period, whereas gain to feed ratio was calculated according to total feed intake and BW gain of each heifer within each period. No treatment × day interaction was detected for hay and hay + concentrate intake (*P *≥ 0.44); hence, feed intake results are reported according to main treatment effect.

No treatment × day, treatment × hour, nor treatment × day × hour interactions were detected (*P *≥ 0.32) for rumen fermentation responses; therefore, results are reported according to main treatment effects (Table 3). Rumen fluid pH and ammonia concentrations were not affected by treatments (*P *≥ 0.49). Inclusion of YC linearly increased (*P *≤ 0.04) propionate and iso-butyrate concentrations in the rumen fluid, and tended (*P *≤ 0.09) to linearly increase acetate and total VFA concentration. Concentrations of butyrate, iso-valerate, valerate, the acetate:propionate ratio, and the proportion of individual VFA according to total VFA were not affected by treatments (*P *≥ 0.12). Values for rumen fluid pH and concentrations of ammonia and VFA on d 0, prior to treatment initiation in each period, are reported in Supplementary Table 1 and were used as covariate for each respective analysis.

**Table 3.** Rumen fermentation responses in rumen-cannulated forage-fed heifers receiving no yeast culture (**YC**) supplementation (**CON**; n = 12), or YC at (as-fed basis) 1.5 g/100 kg of heifer BW (**YC1.5;** n = 12) or 3.0 g/100 kg of heifer body weight (**YC3.0,** n = 12)^1^^,^^2^

					Contrasts (*P*-value)^3^	
Item	CON	YC1.5	YC3.0	SEM	Linear	Quadratic
Rumen fluid pH	6.66	6.64	6.64	0.03	0.50	0.71
Rumen fluid ammonia, mM	1.05	1.04	1.07	0.21	0.55	0.49
Rumen fluid VFA, mM						
Acetate	172.5	177.6	187.3	7.4	0.09	0.77
Propionate	24.0	25.3	25.8	0.9	0.04	0.62
Butyrate	10.1	10.4	10.5	0.4	0.46	0.78
Iso-valerate	1.07	1.07	1.11	0.06	0.59	0.77
Iso-butyrate	1.77	1.85	1.88	0.05	0.03	0.69
Valerate	1.41	1.38	1.43	0.05	0.69	0.43
Acetate:propionate ratio	7.13	6.98	7.22	0.10	0.54	0.14
Total	210	218	228	8	0.08	0.84
Rumen fluid VFA, % of total VFA						
Acetate	81.5	81.3	81.6	0.2	0.37	0.27
Propionate	11.5	11.7	11.4	0.2	0.64	0.12
Butyrate	4.88	4.85	4.72	0.13	0.41	0.75
Iso-valerate	0.512	0.511	0.508	0.031	0.91	0.98
Iso-butyrate	0.855	0.874	0.864	0.021	0.74	0.51
Valerate	0.686	0.655	0.658	0.021	0.25	0.44

^1^Heifers were assigned to a 3 × 3 Latin square design, containing 3 periods of 21 d each and a washout interval of 14 d between periods. The YC contained dead yeast culture of *Saccharomyces cerevisiae* with growing medium and metabolites (Factor SC, GRASP Indústria e Comércio Ltda., Curitiba, Brazil), with 1.9 × 10^9^ dead cells/g of growth medium and no presence of live cells (Dias et al., 2018).

^2^Ruminal fluid samples were collected on d 0, 8, and 16 of each period, immediately prior to (0 h) and at 4, 8, and 12 h relative to concentrate feeding of the day as in Pickett et al. (2022). The pH of the rumen samples was immediately measured using portable a meter (Traceable™; Thermo Fisher Scientific, Waltham, MA). A 5-mL subsample of each rumen fluid sample was transferred into individual falcon tubes containing 1 mL of 25% metaphosphoric acid for analyses of volatile fatty acids (**VFA**) and ammonia content (Cappellozza et al., 2013). Results from d 0 were averaged and used as covariate for each respective analysis. No treatment × day, treatment × hour, nor treatment × day × hour interactions were detected (*P *≥ 0.32) for the parameters reported herein; therefore, results are reported according to main treatment effects.

No treatment × day interaction was detected (*P *≥ 0.27) for microbiota assessment and results are reported according to main treatment effects (Table 4). Relative abundance of individual bacterial phyla and phyla SD were not affected (*P *≥ 0.12) by treatments. Inclusion of YC linearly decreased (*P *= 0.03) the relative abundance of *Succiniclasticum*, and linearly increased (*P *= 0.04) the genera SD. The relative abundances of all other bacterial genera were not affected (*P *≥ 0.12) by treatments. Values for microbiota assessment on d 0 are reported in Supplementary Table 2, and were used as covariate for each respective analysis.

**Table 4.** Bacterial composition (relative abundance, %) and diversity [Shannon diversity (**SD;**Kim et al., 2017)] in the rumen fluid of rumen-cannulated forage-fed heifers receiving no yeast culture (**YC**) supplementation (**CON**; n = 12), or YC at (as-fed basis) 1.5 g/100 kg of heifer BW (**YC1.5;** n = 12) or 3.0 g/100 kg of heifer body weight (**YC3.0,** n = 12). Only bacterial phyla and genera with relative abundance above 1% are reported^1^^,^^2^

					Contrasts (*P*-value)^3^	
Item	CON	YC1.5	YC3.0	SEM	Linear	Quadratic
Bacterial phyla						
Bacteroidetes	57.9	59.1	57.2	1.1	0.67	0.23
Firmicutes	25.2	23.9	24.9	0.7	0.74	0.12
Proteobacteria	9.00	8.85	9.63	0.55	0.44	0.44
Euryarchaeota	2.68	2.81	2.63	0.19	0.81	0.41
Tenericutes	1.24	1.17	1.44	0.16	0.39	0.38
Spirochaetes	1.58	1.62	1.48	0.10	0.45	0.47
SD index	1.21	1.22	1.23	0.01	0.40	0.67
Bacterial genera						
* Prevotella*	29.3	30.6	28.6	0.9	0.53	0.12
* Bacteroides*	18.1	18.3	17.6	0.6	0.48	0.55
* Pedobacter*	4.31	4.13	4.51	0.28	0.53	0.32
* Succiniclasticum*	3.10	2.74	2.40	0.22	0.03	0.98
* Methanobrevibacter*	2.88	3.06	2.86	0.22	0.92	0.37
* Blautia*	2.97	2.81	2.94	0.13	0.78	0.19
* Clostridium*	2.73	2.45	2.72	0.13	0.95	0.12
* Alkaliphilus*	1.72	1.57	1.87	0.23	0.65	0.41
* Ruminococcus*	2.35	2.35	2.55	0.11	0.22	0.45
* Dysgonomonas*	2.06	2.11	2.30	0.21	0.25	0.73
* Caloramator*	2.00	1.86	1.85	0.11	0.19	0.47
* Oscillospira*	1.79	1.56	1.76	0.13	0.80	0.14
* Butyrivibrio*	1.95	1.70	1.74	0.12	0.12	0.19
* Treponema*	1.83	1.88	1.73	0.11	0.55	0.48
* Paludibacter*	1.84	1.89	1.89	0.19	0.87	0.92
* Paraprevotella*	1.37	1.40	1.48	0.11	0.46	0.84
* Porphyromonas*	1.11	1.19	1.25	0.10	0.30	0.92
SD index	2.92	2.93	3.01	0.03	0.04	0.23

^1^Heifers were assigned to a 3 × 3 Latin square design, containing 3 periods of 21 d each and a washout interval of 14 d between periods. The YC contained dead yeast culture of *Saccharomyces cerevisiae* with growing medium and metabolites (Factor SC, GRASP Indústria e Comércio Ltda., Curitiba, Brazil), with 1.9 × 10^9^ dead cells/g of growth medium and no presence of live cells (Dias et al., 2018).

^2^Ruminal fluid samples were collected on d 0, 8, and 16 of each period immediately prior to treatment administration for microbiota analysis as in Pickett et al. (2022). Results from d 0 were used as covariate for each respective phylum or genus. No treatment × day interaction was detected (*P *≥ 0.27) for the parameters reported herein; therefore, results are reported according to main treatment effects.

Rumen liquid volume and dilution rate were not affected (*P *≥ 0.29) by treatments (Table 5). Inclusion of YC linearly increased (*P *≤ 0.05) ruminal disappearance rates of DM and NDF, whereas effective degradability of DM and NDF did not differ (*P *≥ 0.40) among treatments (Table 5). From d 17 to 21 of each period, daily intake of CP, NDF, starch, and lignin were not altered (*P *≥ 0.15) by treatments (Table 6). Inclusion of YC linearly increased (*P *≤ 0.05) the aTTD of starch and NDF, but did not affect (*P *≥ 0.35) the aTTD of DM and CP (Table 6).

**Table 5.** Rumen liquid dilution rate, rumen liquid volume, and ruminal in situ disappearance parameters of bermudagrass hay (*Cynodon dactylon*) in rumen-cannulated forage-fed heifers receiving no yeast culture (**YC**) supplementation (**CON**; n = 12), or YC at (as-fed basis) 1.5 g/100 kg of heifer body weight (**YC1.5;** n = 12) or 3.0 g/100 kg of heifer BW (**YC3.0,** n = 12)^1^

					Contrasts (*P*-value)^3^	
Item	CON	YC1.5	YC3.0	SEM	Linear	Quadratic
Liquid dilution rate,^2^ %/h	8.62	9.13	8.85	0.45	0.60	0.30
Liquid volume,^2^ mL/kg of BW	213	194	196	13	0.29	0.44
Hay disappearance rate,^3^ %/h						
Dry matter	6.38	7.44	7.50	0.34	0.02	0.21
Neutral detergent fiber	4.44	4.72	4.89	0.18	0.05	0.79
Hay effective degradability,^4^ %						
Dry matter	42.7	42.3	42.5	4.8	0.73	0.56
Neutral detergent fiber	53.1	52.8	53.3	3.8	0.59	0.40

^1^Heifers were assigned to a 3 × 3 Latin square design, containing 3 periods of 21 d each and a washout interval of 14 d between periods. The YC contained dead yeast culture of *Saccharomyces cerevisiae* with growing medium and metabolites (Factor SC, GRASP Indústria e Comércio Ltda., Curitiba, Brazil), with 1.9 × 10^9^ dead cells/g of growth medium and no presence of live cells (Dias et al., 2018).

^2^Liquid dilution rate and volume evaluated via intra-ruminal Co-EDTA pulse-dose on d 17 of each period (prior to concentrate feeding), and rumen fluid collected at 0, 2, 4, 6, 8, 12, 24, 36, 48, 72, and 96 h relative to Co-EDTA infusion (Cappellozza et al., 2013).

^3^Hay disappearance rate was evaluated by suspending Dacron bags containing 4 g of hay on d 17 of each period (prior to concentrate feeding), and incubating by 0, 2, 4, 6, 8, 12, 24, 36, 48, 72, and 96 h.

^4^Effective degradability was calculated by fixing ruminal passage rate at 0.035/h (Scarbrough et al., 2006) and the model proposed by Ørskov and McDonald (1979).

**Table 6.** Nutrient intake and apparent total-tract digestibility in rumen-cannulated forage-fed heifers receiving no yeast culture (**YC**) supplementation (**CON**; n = 12), or YC at (as-fed basis) 1.5 g/100 kg of heifer body weight (**YC1.5;** n = 12) or 3.0 g/100 kg of heifer BW (**YC3.0,** n = 12)^1^^,^^2^

					Contrasts (*P*-value)^3^	
Item	CON	YC1.5	YC3.0	SEM	Linear	Quadratic
Nutrient intake, kg/d						
Crude protein	0.532	0.551	0.535	0.010	0.82	0.15
Acid detergent fiber	1.54	1.61	1.56	0.04	0.82	0.15
Neutral detergent fiber	3.09	3.22	3.11	0.07	0.82	0.15
Starch	0.498	0.508	0.500	0.005	0.82	0.15
Lignin	0.231	0.241	0.233	0.005	0.82	0.15
Apparent total-tract digestibility, % of intake						
Dry matter	58.6	59.3	59.6	1.6	0.26	0.75
Crude protein	60.2	60.8	60.0	1.5	0.79	0.30
Neutral detergent fiber	60.6	61.4	62.5	1.6	0.05	0.83
Starch	91.8	92.9	94.2	0.7	< 0.01	0.98

^1^Heifers were assigned to a 3 × 3 Latin square design, containing 3 periods of 21 d each and a washout interval of 14 d between periods. The YC contained dead yeast culture of *Saccharomyces cerevisiae* with growing medium and metabolites (Factor SC, GRASP Indústria e Comércio Ltda., Curitiba, Brazil), with 1.9 × 10^9^ dead cells/g of growth medium and no presence of live cells (Dias et al., 2018).

^2^Apparent total-tract digestibility of nutrients was calculated by collecting fecal grab samples collected at 12-h intervals beginning on d 17 prior to concentrate (h 0) feeding until d 21 (h 96), according to Ferrareto et al. (2015). Nutrient intake was calculated based on hay and concentrate intake from d 17 to 21, and the nutritional analysis of all feed ingredients.

## DISCUSSION

This metabolism trial was designed to evaluate the effects of YC supplementation to growing beef cattle consuming a forage-based diet, and replicated growth rates typical of stocker cattle (Reuter and Beck, 2013; Mackey et al., 2024). Based on feed intake and YC inclusion rates, heifers assigned to YC1.5 consumed 0.545 g of YC/kg of diet DM whereas YC3.0 consumed 1.12 g of YC/kg of diet DM. Supplementing YC did not impact feed intake but improved heifer ADG in a linear manner, resulting in greater G:F according to YC inclusion. These ADG and G:F results, however, should be interpreted with caution given the 21-d periods and Latin square design utilized herein (Thallman et al., 2018). For that reason, ADG and G:F results are being presented but are not being further discussed in this manuscript.

Batista et al. (2022) compiled a meta-analysis of yeast products for feedlot cattle that included studies with YC and live yeast, although most studies evaluated YC. These authors reported increased growth performance in cattle supplemented with yeast products. Batista et al. (2022) attributed these outcomes to improved digestibility of DM, CP, NDF, and ruminal pH stability, but not to rumen fermentation responses including VFA production. In a meta-analysis with dairy ruminants (Desnoyers et al., 2009), yeast supplementation increased feed intake as the proportion of concentrate in the diet increased. Desnoyers et al. (2009) also suggested that yeast supplementation improved ruminal VFA production in ruminants fed high-forage diets but not in those fed high-grain diets, given the benefits of yeast products to ruminal cellulolysis (Harrison et al., 1988; Callaway and Martin, 1997). Results from Desnoyers et al. (2009) support the findings from the current experiment, in which heifers consumed a high-forage diet (~88:12 forage to concentrate ratio; Table 1) with a C4 grass containing 70% NDF (DM basis).

Inclusion of YC increased ruminal propionate, iso-butyrate, and acetate concentrations that were mainly driven by increased total VFA concentrations; the latter two variables as statistical tendencies. Accordingly, ruminal VFA profile was not altered by treatments when analyzed as proportion of each individual VFA according to total VFA. Therefore, YC supplementation favored ruminal VFA production despite similar feed intake, suggesting greater ability of rumen microbes to utilize and synthesize VFA from dietary nutrients (Rett et al., 2020). Ruminal pH was above 6.64 across treatments and near neutrality, as expected from a high-forage diet (Dijkstra et al., 2012). For this reason, YC supplementation did not affect ruminal pH herein because its buffering capacity is mostly expressed in acidotic conditions (Desnoyers et al., 2009; Batista et al., 2022). The minimum pH value noted during this experiment across all heifers was 6.19; hence, treatment effects on minimum pH or proportion of time in acidotic conditions were not investigated (Dijkstra et al., 2012; Vyas et al., 2014). The YC effects on ruminal VFA production and profile were also not sufficient to affect ruminal pH, and suggest a prebiotic benefit to ruminal microbes (Oeztuerk et al., 2005). Concentrations of ammonia in the rumen fluid were not affected by YC supplementation, as in the meta-analysis by Batista et al. (2022) and previous studies (Moya et al., 2009; Vyas et al., 2014). Others have reported decreased ruminal ammonia concentration in cattle supplemented with YC or other yeast products, although such responses were relatively small and with limited biological significance (Wallace and Newbold, 1995; Baker et al., 2022).

The most common phyla in the rumen were Bacteroidetes and Firmicutes as previously documented (Pickett et al., 2022), with Bacteroidetes including bacteria responsible for degrading structural carbohydrates (Petri et al., 2013; Ramos et al., 2021). The most abundant genus in the rumen fluid was *Prevotella* (Pickett et al. 2022), which degrades starch, β glycans, protein, pectin, and hemicellulose in the rumen (Henderson et al., 2015). Inclusion of YC did not affect the relative abundance of the bacterial phyla nor phyla alpha-diversity, and linearly decreased the relative abundance of *Succiniclasticum*. This genus is known to convert succinate into propionate (van Gylswyk, 1995), which is contrary to the YC effects noted for propionate concentrations in the rumen fluid. In turn, SD bacterial genera increased according to the level of YC inclusion, denoting an increase in genera alpha-diversity from YC supplementation (Kim et al., 2017). Nonetheless, the overall effects and biological implications from YC supplementation on rumen bacterial composition were relatively small, indicating the YC did not alter the ruminal microbiota profile of cattle consuming high-forage diets herein.

Based on rumen microbiota results, YC inclusion improved VFA production by enhancing the ability of existing rumen microbes in utilizing dietary nutrients, which corroborates the prebiotic activity of YC (Oeztuerk et al., 2005; Newbold and Rode, 2006). The in situ disappearance rate of dietary hay supports this rationale, suggesting greater forage degradability in the rumen according to YC inclusion (Nocek et al., 1979). Treatment differences on hay disappearance, however, were not sufficient to alter the estimated effective rumen degradability based on the Ørskov and McDonald (1979) model, and using a ruminal passage rate of 0.035/h. Effective rumen degradability can be calculated by fixing different ruminal passage rates (i.e., 0.020, 0.046, or 0.060/h; Silva et al., 2024), and the value used herein was specific to bermudagrass (Scarbrough et al., 2006).

Rumen liquid dilution rate and liquid volume are directly associated with voluntary feed intake (Allison, 1985; Allen, 2000), whereas liquid dilution reflects ruminal motility and passage of solubilized ruminal substrates (Waggoner et al., 2009). None of these parameters were affected by YC inclusion, which does not help addressing the disconnection between the YC effects on hay effective degradability and rumen disappearance rate. Alternatively, the increased aTTD of NDF according to YC inclusion indicates that rumen degradability of this nutrient was improved by YC supplementation (Huhtanen et al., 2010; Ferraretto et al., 2015). Despite the diet used herein having limited starch content, the increased aTTD of starch also denotes greater ruminal degradability, and perhaps increased postruminal digestibility of this nutrient according to YC inclusion (Ørskov, 1986).

In summary, results from this experiment support our main hypothesis as YC improved rumen fermentation responses of beef heifers consuming a forage-based diet. The treatment differences observed for ruminal disappearance rate of hay DM and NDF, as well as aTTD of NDF and starch provides evidence of enhanced nutrient utilization by ruminal microbes, leading to improved ruminal VFA production according to YC inclusion level. Research is still warranted to further investigate the biological and productive benefits of YC supplementation to forage-fed growing cattle, including longer supplementation periods (i.e., 90 to 120 d) to properly evaluate growth performance relevant to stocker and other grazing production systems (Reuter and Beck, 2013; Mackey et al., 2024).

## Supplementary Material

txaf103_suppl_Supplementary_Materials_1
