# Supplementary material for: Supplementing yeast culture to beef heifers consuming a forage-based diet
Source: Transl Anim Sci. 2025 Aug 19;9:txaf103. doi: 10.1093/tas/txaf103 (PMC12416140; doi:10.1093/tas/txaf103)
Supplement: txaf103_suppl_Supplementary_Materials_1 [file txaf103_suppl_supplementary_materials_1.docx]

**Supplementary Table 1.** Rumen fermentation responses in rumen-cannulated forage-fed heifers prior to treatment application.^1^

|  |  |  |  |  |  | **Contrasts (*P*-value)**^3^ | |
| --- | --- | --- | --- | --- | --- | --- | --- |
| **Item** | **CON** | **YC1.5** | **YC3.0** | **SEM** |  | **Linear** | **Quadratic** |
| Rumen fluid pH | 6.55 | 6.56 | 6.54 | 0.04 |  | 0.82 | 0.58 |
| Rumen fluid ammonia, mM | 1.01 | 1.00 | 1.02 | 0.25 |  | 0.72 | 0.55 |
| Rumen fluid VFA, mM | | |  |  |  |  |  |
| Acetate | 170 | 170 | 177 | 11 |  | 0.19 | 0.45 |
| Propionate | 25.7 | 25.9 | 27.0 | 1.4 |  | 0.44 | 0.75 |
| Butyrate | 10.5 | 9.84 | 10.2 | 0.65 |  | 0.71 | 0.39 |
| Iso-valerate | 1.34 | 1.27 | 1.32 | 0.08 |  | 0.79 | 0.49 |
| Iso-butyrate | 1.91 | 1.88 | 2.11 | 0.14 |  | 0.34 | 0.46 |
| Valerate | 1.32 | 1.27 | 1.42 | 0.06 |  | 0.22 | 0.14 |
| Acetate:propionate ratio | 6.63 | 6.69 | 6.68 | 0.19 |  | 0.83 | 0.87 |
| Total | 211 | 210 | 219 | 13 |  | 0.23 | 0.46 |
| Rumen fluid VFA, % of total VFA | | | | |  |  |  |
| Acetate | 80.1 | 80.7 | 80.6 | 0.4 |  | 0.34 | 0.42 |
| Propionate | 12.3 | 12.3 | 12.2 | 0.3 |  | 0.95 | 0.88 |
| Butyrate | 5.16 | 4.73 | 4.60 | 0.22 |  | 0.07 | 0.57 |
| Iso-valerate | 0.750 | 0.660 | 0.767 | 0.040 |  | 0.72 | 0.02 |
| Iso-butyrate | 1.00 | 0.943 | 1.09 | 0.06 |  | 0.18 | 0.11 |
| Valerate | 0.651 | 0.618 | 0.666 | 0.026 |  | 0.62 | 0.13 |

^1^ Heifers were assigned to a 3 × 3 Latin square design, containing 3 periods of 21 d each and a washout interval of 14 d between periods. Rumen fluid was collected on d 0, immediately prior to (0 h) and at 4, 8, and 12 h relative to concentrate feeding of the day (Pickett et al., 2022). The pH of the rumen samples was immediately measured using portable a meter (Traceable™; Thermo Fisher Scientific, Waltham, MA). A 5-mL subsample of each rumen fluid sample was transferred into individual falcon tubes containing 1 mL of 25% metaphosphoric acid for analyses of volatile fatty acids (**VFA**) and ammonia content (Cappellozza et al., 2013). Heifers were assigned to receive no yeast culture (**YC**) supplementation (**CON**; n = 12), or YC at (as-fed basis) 1.5 g/100 kg of heifer BW (**YC1.5;** n = 12) or 3.0 g/100 kg of heifer body weight (**YC3.0,** n = 12). No treatment × hour interactions were detected (*P* ≥ 0.41) for the parameters reported herein; hence, results are reported according to main treatment effects.

**Supplementary Table 2.** Bacterial composition (relative abundance, %) and diversity [Shannon diversity (**SD;** Kim et al., 2017)] in the rumen fluid of rumen-cannulated forage-fed heifers prior to treatment application. Only bacterial phyla and genera with relative abundance above 1% are reported.^1^

|  |  |  |  |  |  | **Contrasts (*P*-value)**^3^ | |
| --- | --- | --- | --- | --- | --- | --- | --- |
| **Item** | **CON** | **YC1.5** | **YC3.0** | **SEM** |  | **Linear** | **Quadratic** |
| Bacterial phyla |  |  |  |  |  |  |  |
| Bacteroidetes | 56.3 | 57.2 | 58.1 | 1.1 |  | 0.21 | 0.95 |
| Firmicutes | 26.4 | 26.8 | 27.1 | 0.8 |  | 0.48 | 0.96 |
| Proteobacteria | 8.17 | 7.51 | 6.78 | 0.36 |  | 0.01 | 0.92 |
| Euryarchaeota | 4.20 | 3.47 | 3.37 | 0.55 |  | 0.06 | 0.35 |
| Tenericutes | 1.02 | 1.06 | 1.20 | 0.10 |  | 0.24 | 0.68 |
| Spirochaetes | 1.54 | 1.61 | 1.45 | 0.15 |  | 0.63 | 0.44 |
| SD index | 1.25 | 1.21 | 1.20 | 0.02 |  | 0.03 | 0.38 |
| Bacterial genera |  |  |  |  |  |  |  |
| *Prevotella* | 27.9 | 29.9 | 29.4 | 1.4 |  | 0.45 | 0.42 |
| *Bacteroides* | 16.7 | 17.0 | 17.5 | 0.7 |  | 0.38 | 0.93 |
| *Pedobacter* | 5.13 | 4.56 | 4.76 | 0.26 |  | 0.26 | 0.18 |
| *Succiniclasticum* | 3.92 | 4.38 | 4.63 | 0.38 |  | 0.16 | 0.79 |
| *Methanobrevibacter* | 4.55 | 3.72 | 3.56 | 0.58 |  | 0.02 | 0.33 |
| *Blautia* | 2.80 | 2.73 | 2.51 | 0.16 |  | 0.18 | 0.69 |
| *Clostridium* | 2.65 | 2.65 | 2.61 | 0.07 |  | 0.70 | 0.77 |
| *Alkaliphilus* | 1.42 | 1.53 | 1.56 | 0.16 |  | 0.55 | 0.84 |
| *Ruminococcus* | 2.39 | 2.26 | 2.79 | 0.36 |  | 0.42 | 0.43 |
| *Dysgonomonas* | 2.75 | 2.18 | 2.30 | 0.21 |  | 0.14 | 0.18 |
| *Caloramator* | 2.26 | 2.35 | 2.41 | 0.15 |  | 0.51 | 0.95 |
| *Oscillospira* | 2.27 | 1.78 | 2.01 | 0.21 |  | 0.40 | 0.16 |
| *Butyrivibrio* | 2.12 | 1.99 | 2.01 | 0.12 |  | 0.50 | 0.55 |
| *Treponema* | 1.76 | 1.83 | 1.65 | 0.16 |  | 0.59 | 0.44 |
| *Paludibacter* | 1.26 | 1.38 | 1.35 | 0.19 |  | 0.75 | 0.73 |
| *Paraprevotella* | 1.13 | 1.04 | 1.16 | 0.12 |  | 0.88 | 0.46 |
| *Porphyromonas* | 1.71 | 1.32 | 1.02 | 0.18 |  | 0.01 | 0.81 |
| SD index | 3.00 | 2.94 | 2.96 | 0.04 |  | 0.53 | 0.42 |

^1^ Heifers were assigned to a 3 × 3 Latin square design, containing 3 periods of 21 d each and a washout interval of 14 d between periods. Rumen fluid was collected on d 0 for microbiota analysis as in Pickett et al. (2022), prior the beginning of treatment administration. Heifers were assigned to receive no yeast culture (**YC**) supplementation (**CON**; n = 12), or YC at (as-fed basis) 1.5 g/100 kg of heifer BW (**YC1.5;** n = 12) or 3.0 g/100 kg of heifer body weight (**YC3.0,** n = 12).
